# Insect Oil as An Alternative to Palm Oil and Poultry Fat in Broiler Chicken Nutrition

**DOI:** 10.3390/ani9030116

**Published:** 2019-03-25

**Authors:** Abdelbasset Benzertiha, Bartosz Kierończyk, Mateusz Rawski, Paweł Kołodziejski, Magdalena Bryszak, Damian Józefiak

**Affiliations:** 1 Department of Animal Nutrition, Poznań University of Life Sciences, Wołyńska 33, 60-637 Poznań, Poland; basset@up.poznan.pl or abdelbasset.benzertiha@hipromine.com (A.B.); bartosz.kieronczyk@up.poznan.pl (B.K.); magdalena.bryszak@up.poznan.pl (M.B.); 2 HiProMine S.A., Poznańska 8, 62-023 Robakowo, Poland; 3 Institute of Zoology, Division of Inland Fisheries and Aquaculture, Poznań University of Life Sciences, Wojska Polskiego 71c, 60-625 Poznań, Poland; mateusz.rawski@up.poznan.pl; 4 Department of Animal Physiology and Biochemistry, Poznań University of Life Sciences, Wołyńska 33, 60-637 Poznań, Poland; pawelbigi@o2.pl

**Keywords:** insect oil, *Tenebrio molitor*, mealworm, broiler chicken, palm oil, poultry fat

## Abstract

**Simple Summary:**

Recently, there has been increasing interest in the use of insects as an alternative sustainable source of protein and fat in animal feed to improve animal production and maintain ecological sustainability. Palm oil is commonly used in broiler chicken nutrition; however, due to the environmental footprint, consumers have formed negative opinions regarding its applications. Therefore, alternatives to palm oil are urgently needed. The present study was conducted to evaluate the effects of *Tenebrio molitor* oil as a total replacement for palm oil and poultry fat in broiler chicken diets on chicken performance, nutrient digestibility, pancreatic enzyme activity, various blood parameters and lipid fatty acid compositions of liver and breast muscle tissues. Based on the obtained results, *T. molitor* oil did not show any adverse impacts on performance and improved the fatty acid profiles of liver and breast muscle tissues. In conclusion, *T. molitor* oil may be a sustainable alternative to palm oil in broiler chicken nutrition.

**Abstract:**

This study was conducted to evaluate the effects of *Tenebrio molitor* (TM) oil as a total replacement for palm oil and poultry fat in broiler chicken diets on growth performance, nutrient digestibility, pancreatic enzyme activity, selected blood parameters and the lipid fatty acid compositions of liver and breast muscle tissues. A total of 72 seven-day-old female Ross 308 broiler chickens were used. The birds were randomly distributed into three groups with 12 replicates each, using two birds per replicate for 30 days in metabolic cages. The basal diet was supplemented with 5% palm oil, poultry fat or TM oil. There was no effect (*p* > 0.05) caused by the dietary oil replacement on the birds’ performance and apparent nutrient digestibility. Liver size (*p* = 0.033), the concentration of hepatic triglycerides (*p* = 0.049) and total cholesterol (*p* = 0.048) were reduced by TM oil supplementation. Furthermore, TM oil supplementation increased n-3 and n-6 fatty acids (*p* = 0.006; *p* < 0.001, respectively) in breast muscle tissue. In conclusion, the use of TM oil in broiler chickens’ diets did not show any adverse effects on performance, nutrient digestibility and blood biochemical parameters. Moreover, TM oil supplementation improved the fatty acid profiles of liver and breast muscle tissues.

## 1. Introduction

The cost and the demand to increase the energy value of feed diets to meet the requirements of fast-growing birds have become a subject of interest for nutritionists. The supplementation of dietary fats is one of the preferred methods to achieve this purpose [1,2]. Various fat sources have been introduced in poultry nutrition, such as rendering by-products (e.g., lard, tallow and poultry fat) and vegetable oils (e.g., soybean, maize, palm, colza, linseed, rapeseed, sesame seed, sunflower seed and pumpkin seed) [1]. Recently, insects have been proposed as a sufficient, sustainable and alternative source of fat and protein for poultry [3,4,5]. With an acceptable amount of fat, insects may replace other fat sources without any adverse effects on growth performance, the lipid fatty acid compositions of liver and breast muscle tissue, intestinal morphology or histological features [6,7,8]. The abovementioned studies highlighted the use of three insect species, i.e., *Tenebrio molitor* (mealworm), *Zophobas morio* (super worm) and *Hermetia illucens* (black soldier fly, BSF), for a total or partial replacement of soybean oil. Palm oil (PO), which comes from the fruit of the *Elaeis guineensis* tree, is the second most common vegetable oil produced globally following soybean oil. According to the United States Department of Agriculture (USDA), its worldwide production was estimated to be 70.37 million metric tons in 2017/2018 [9]. Tan Choon-Hui et al. [10] showed that the major saturated fatty acids (SFAs) in PO were palmitic (43.7%) and stearic (4.5%) acids, and the main unsaturated fatty acids (UFAs) were oleic (40.2%) and linoleic (9.6%) acids. PO is widely used in human and animal nutrition [11]. According to Skřivan et al. [11], palm oil is a common supplement in poultry diets. However, due to the negative impact on the environment, i.e., deforestation, extinction of endangered species and loss of biodiversity, as well as nutritional issues (high ratio of saturated fatty acids), consumers are looking for an alternative energy source [11]. One solution could be the use of *T. molitor* larvae, which are a source of protein (451–603 g/kg of DM), as well as fat (250–431 g/kg of dry matter content) [5]. The fatty acid (FA) profile of *T. molitor* is dominated by oleic and linoleic acids [8,12,13]. Almost all insects can biosynthesize palmitic, stearic and oleic acids. In addition, the profile and content of FAs in insects depend on various parameters, such as diet, species, environment and life stage [12,14]. In the available literature, there are no data concerning the replacement of palm oil and poultry fat (PF) with insect oil in broiler chicken diets. Therefore, the present study was conducted to evaluate the effect of *T. molitor* oil (TM oil) as a total replacement for palm oil and poultry fat in broiler chicken diets on their growth performance, nutrient digestibility, pancreatic enzyme activity, various blood parameters and lipid fatty acid compositions of liver and breast muscle tissues.

## 2. Material and Methods

### 2.1. Birds and Experimental Design

The experimental protocols were in accordance with guidelines and were approved by the Local Ethics Commission of the Poznań University of Life Sciences, Poznań, Poland (Authorization No. 8/2015). All efforts were made to minimise animals suffering.

A total of 72 seven-day-old female Ross 308 chicks were used. The birds were weighed and randomly distributed into 3 different groups. A total of 12 replicates per treatment were used with 2 birds per replicate. The birds were kept in metabolic cages until they reached 30 days of age. The housing conditions were in accordance with the Aviagen recommendations. The temperature was maintained at 27 °C until day 7 and was gradually reduced to 21 °C by day 21; thereafter, it was kept constant. The lighting cycles (hours of light/hours of dark) were 19:5 from day 7 to 21 and 23:1 from day 22 to 30.

The composition of the chickens’ basal diet is shown in Table 1. All birds had ad libitum access to water and feed for the whole period of the experiment. The basal diet was designed according to Tancharoenrat et al. [15] and formulated on a maize and soybean meal base to meet or exceed the daily nutrient requirements of broiler chickens. Then, 5% poultry fat, palm oil or *T. molitor* oil was added to the basal diet. A total of 3 experimental treatments were used and were designed as follows: poultry fat (PF)—5% poultry fat added to the basal diet; palm oil (PO)—5% palm oil added to the basal diet; and *T. molitor* oil (TM)—5% *Tenebrio molitor* oil added to the basal diet. The diets were formulated and produced in a pellet form and crumbled at the Piast Pasze factory (Lewkowiec, Poland) according to the international organization for standardization (ISO) 9001:2008 procedures. The birds were fed one type of diet from the start to the end of the experiment (days 7–30 of age); the diets differed only in the source of fat. No additives were added to the diets. Throughout the entire experiment, 0.3% titanium dioxide (TiO_2_) was added as an internal marker for the calculation of nutrient digestibility. 

### 2.2. Sample Collection

The birds were weighed at days 7, 14, 21 and 30. The body weight gain (BWG) and feed intake (FI) were measured, and the feed conversion ratio (FCR) was calculated for days 7–14, 14–21, 21–30 and 7–30. At the end of the experiment (day 30), the birds were sacrificed by cervical dislocation. Then, 12 birds per treatment were randomly selected, and blood samples were collected after decapitation. Serum was obtained by centrifugation (Micro 220R, Hettich, Tuttlingen, Germany) at 1000× *g* at 8 °C for 10 min and stored at *−*20 °C until analysis. The following blood parameters were determined: non-esterified fatty acid (NEFA) content, glucose, triglycerides (TG), total cholesterol (TCh), total protein (TP), albumin and insulin. The crop, jejunum and ceca were emptied by gentle pressure; digesta were collected for pH measurements. The contents of the ileum between the Meckel’s diverticulum and the ileo–cecal–colonic junction were collected for apparent ileal digestibility of crude protein and crude fat, as well as apparent metabolizable energy corrected to zero nitrogen balance (AME_N_). The duodenal content was collected into sterile plastic bags and stored at −80 °C for pancreatic enzyme analysis. Immediately after slaughter, breast muscle and liver tissue were cut, directly packed in flexi grip bags and put onto dry ice to await further analyses.

### 2.3. Analyses of Selected Serum Parameters and Pancreatic Enzymes

The concentration of non-esterified fatty acids NEFA was measured according to the colorimetric method described by Duncombe [16]. Glucose concentrations were analysed calorimetrically using a Synergy 2 Multi-Mode Reader (BioTek Instruments, Inc., Winooski, VT, USA) with a kit reagent (Pointe Scientific, Warsaw, Poland). Insulin concentrations were determined using commercial radioimmunoassay kits (Merck Millipore, Darmstad, Germany) according to the manufacturer’s instructions. TCh and TG were measured using commercial enzymatic kits (Pointe Scientific, Warsaw, Poland). TP and albumin were determined according to the methods described by Szymeczko et al. [17].

The pancreatic enzyme activity was measured as described in detail by Pruszyńska-Oszmalek et al. [18]. In brief, 250 μL of phosphate buffer solution (PBS) was added to 100 mg of content, and the mixture was homogenised on ice and centrifuged (10,000× *g* for 16 min). The activity of lipase, amylase and trypsin of the duodenal digesta was measured using appropriate commercial colorimetric assay kits (BioVision, Milpitas, CA, USA).

### 2.4. Selected Fat Sources

Insect fat (*T. molitor* oil) was obtained from a commercial source (HiProMine S.A., Robakowo, Poland), while poultry fat and palm oil were purchased from a different commercial source (Piast Pasze Sp. z o.o., Lewkowiec, Poland). The fatty acid composition and profile of the abovementioned fats are presented in Table 2. Dietary insect fat was extracted using supercritical CO_2_ according to Jackowski et al. [19]. Briefly, before the extraction, materials were air dried in an oven (SLN 240, POL-EKO Aparatura, Wodzisław Śląski, Poland) for 24 h at 50 °C. Then, an extraction was performed at a pressure of 300 bar and a temperature of 40 °C. For extract collection, a two-separator system was used. The CO_2_ flow was adjusted to 110.4 kg/h. Commercial CO_2_ (99% purity, Zakłady Azotowe, Puławy, Poland) was used for the extraction. 

### 2.5. Chemical Analysis

The nutrient content of the diets and digesta were analysed according to the association of official analytical chemists (AOAC) [20] using the 934.01, 984.13 and 920.39 methods for dry matter (DM), crude protein (CP) and crude fat (CF), respectively. TiO_2_ analysis was determined according to Myers et al. [21], and the concentration was estimated as described by Short et al. [22]. Gross energy (GE) was analysed using an adiabatic bomb calorimeter (KL 12 Mn, Precyzja-Bit PPHU Sp. z o.o., Bydgoszcz, Poland) standardised with benzoic acid.

Lipids from the liver and breast tissues were saponified according to the procedure described by Głogowski et al. [23]. Briefly, the tissues were homogenised and treated with a mixture of 1 mL 2 M KOH in water and 1 mL 1 M KOH in methanol. The samples were vortexed and heated to 95 °C for 10 min, cooled at room temperature for 10 min and sonicated for 10 min. The mixture was protected from the light and kept overnight in a screw-capped Teflon-stoppered tube under nitrogen at 23 °C. Afterwards, 1.5 mL of water was added to the mixture, and the obtained solution was acidified by 4 M HCl to a pH below 2. The extraction procedure was repeated 4 times using diethyl ether. The extracted FAs were esterified using 0.5 M NaOH in methanol and subsequently converted to fatty acid methyl esters (FAME) using methanol boron trifluoride (Fluka, Sigma-Aldrich, Steinheim, Germany).

FAME in the PF, PO and TM oil, as well as in the breast muscle and liver tissues, were determined by gas chromatography according to Cieślak et al. [24]. Briefly, a gas chromatograph (GC Bruker 456-GC, Billerica, MA, USA) equipped with a capillary column (100 m fused-silica, 0.25 mm i.d., 0.25 µm film thickness; Chrompack CP7420, Agilent HP) and a flame ionisation detector was injected with 1 μL of sample into the column. Hydrogen was used as the carrier gas at a flow rate of 1.3 mL/min. FAs were determined based on their retention times and were expressed as the proportion of the sum of identified FAs (g/100 g FA). Peaks were identified by comparing retention times with appropriate fatty acid methyl ester standards (37 FAME Mix, Supelco, Poole, UK) using Galaxie Work Station 10.1 (Varian, Walnut Creek, CA, USA). The atherogenic index [25] and thrombogenic index [26] values were calculated using the appropriate equations.

### 2.6. Calculations

The internal organ indexes were calculated as the organ weight (g) divided by the live chicken weight [27].

The apparent ileal digestibility of crude protein and crude fat were calculated relative to the ratio of TiO_2_ (dietary marker) to the nutrient content in feed or digesta. The relative N retention coefficient was calculated as described by Kaczmarek et al. [28]. The following equation was used (crude protein digestibility calculation as an example):

(1) $$D_{\mathrm{crude}\;\mathrm{protein}}=1-[\left(\frac{{\mathrm{TiO}}_{2\frac{g}{\mathrm{kg}\;\mathrm{diet}}}}{{\mathrm{TiO}}_{2\frac{g}{\mathrm{kg}}\mathrm{digesta}}}\right)\times (\frac{{\mathrm{Crude}\;\mathrm{protein}}_{\frac{g}{\mathrm{kg}}\mathrm{digesta}}}{{\mathrm{Crude}\;\mathrm{protein}}_{\frac{g}{\mathrm{kg}}\mathrm{diet}}})]$$

### 2.7. Statistical Analysis

The experiments had a completely randomised design. All data were tested for normal distributions using the Kolmogorov–Smirnov test. An analysis of variance was conducted using Bartlett’s test. The significance of differences among the groups was determined with Duncan’s multiple range test at the significance level of *p* < 0.05. The analyses were performed using SAS software (SAS Institute Inc., Cary, NC, USA). In the experiment, the following general model was used:

(2) $$Y_{i}=\mu +\alpha_{i}+\delta_{ij}$$

where *Y_i_* is the observed dependent variable, *μ* is the overall mean, *α_i_* is the effect of dietary fat source and *δ_ij_* is the random error.

## 3. Results

### 3.1. Fatty Acid Profiles of the Fat Sources

The fatty acid profiles of the fat sources used in the present study are shown in Table 2. Unsaturated fatty acids (both MUFAs and PUFAs) were dominant in *T. molitor* oil and poultry fat. In contrast, the highest concentration of SFAs was present in palm oil. The most dominant UFAs were oleic and linoleic in all fat sources. Compared with palm oil and poultry fat, *T. molitor* oil had the highest concentration of PUFAs and MUFAs and the lowest medium-chain fatty acids (MCFAs). In addition, the SFA value was the lowest in TM oil (21.6%), followed by PF (29.92%) and PO (44.98%), respectively.

### 3.2. Growth Performance

The growth performance results are shown in Table 3. No significant (*p* > 0.05) differences among treatments were observed in each experimental period, i.e., 7–14, 15–21, 22–30, and 7–30 days old, in the case of BWG, FI and FCR.

### 3.3. Weight of Internal Selected Organs

The weights of the internal selected organs are shown in Table 4. Significant differences in the relative weight of the liver were observed (*p* = 0.033). *T. molitor* oil reduced the size of the liver in comparison with poultry fat. Pancreas size was not affected by any dietary treatment (*p* = 0.571).

### 3.4. Activity of Endogenous Enzymes

Endogenous enzyme activity is shown in Table 5. Dietary treatments did not influence lipase (*p* = 0.695) and trypsin (*p* = 0.197) activity. However, amylase showed decreased activity with TM oil supplementation in comparison with the PF treatment (*p* = 0.034). 

### 3.5. Gastrointestinal Tract pH and Apparent Ileal Digestibility

The pH values of the crop, jejunum and cecal contents were not affected (*p* > 0.05) by any of the dietary treatments (Table 6). The apparent ileal digestibility of crude protein, crude fat and AME_N_ is shown in Table 7. No statistically significant differences (*p* > 0.05) were found in the digestibility of crude protein or crude fat or in the AME_N_.

### 3.6. Selected Blood and Liver Parameters

The concentrations of the selected blood parameters are shown in Table 8. The TG values showed statistically significant differences (*p* = 0.040), and TM oil supplementation decreased the level of TG in comparison with the PF treatment. In addition, the dietary treatment did not affect the level of other parameters (i.e., TCh, NEFA, TP, albumin, glucose and insulin). 

The concentration of the selected lipid parameters and glycogen in the liver is shown in Table 9. TG and TCh were significantly affected by the dietary treatments (*p* = 0.049 and *p* = 0.048, respectively). The levels off TG and TCh were reduced in the case of TM oil supplementation. However, glycogen levels were not affected by any treatment (*p* = 0.337). 

### 3.7. Fatty Acid Profiles in the Liver and Breast Muscle

The effect of the dietary treatments on the fat content and the FA profiles of the liver are summarised in Table 10 and Table 11. The liver tissue of the chickens fed a diet supplemented with TM oil showed the lowest values of SFAs (*p* = 0.004) and the highest values of UFAs (*p* = 0.004). In addition, TM oil supplementation significantly decreased MUFAs (*p* < 0.001) and increased PUFAs (*p* < 0.001) in comparison with the PF and PO treatments. Moreover, n-6 fatty acids (*p* < 0.001) and n-3 fatty acids (*p* < 0.001) were increased significantly by TM oil supplementation. Simultaneously, the ratio of n-6/n-3 was significantly lowered (*p* = 0.013) by TM oil supplementation in comparison with the other treatments. The liver tissue of the chickens fed the diet supplemented with TM oil had a higher PUFA/SFA ratio (*p* < 0.001). MCFAs were significantly decreased and LCFAs were increased by TM oil supplementation (*p* < 0.001). Thrombogenic and atherogenic indexes were also significantly lowered by TM oil supplementation in comparison with the PF- and PO-supplemented groups (*p* < 0.001).

The total lipids of the liver were significantly reduced in the case of TM oil and PO supplementation (*p* = 0.013). In addition, significant changes were observed in most SFAs, such as C12:0 (*p* = 0.001), C14:0 (*p* = 0.016), C15:0 (*p* < 0.001), C16:0 (*p* < 0.001), C17:0 (*p* < 0.001), C18:0 (*p* < 0.001), C20:0 (*p* = 0.003), C21:0 (*p* < 0.001), C22:0 (*p* < 0.001) and C24:0 (*p* < 0.001); TM oil showed the lowest values for C:14 and C:16. Furthermore, significant differences were observed in most MUFAs; TM oil showed the highest values for C15:1 (*p* = 0.013), C17:1 (*p* < 0.001), C20:1 *trans* (*p* < 0.001) and C24:1 (*p* < 0.001). PUFAs were also affected by the dietary treatments, and TM oil showed the highest values for C18:2 c9c12 (*p* < 0.001), C18:3 c9c12c15 (*p* < 0.001), C20:3 n-6 (*p* < 0.001), C20:4 n-6 (*p* < 0.001), C20:5 n-3 (*p* < 0.001) and C22:5 n-3 (*p* < 0.001).

The FA compositions of the breast muscle tissues are shown in Table 12 and Table 13. The fat content of the breast was not affected (*p* > 0.05). However, significant differences were registered for most SFAs. TM oil supplementation decreased the content of C12:0 (*p* < 0.001), C16:0 (*p* < 0.001), C18:0 (*p* < 0.001) and C23:0 (*p* = 0.002) and increased C14:0 (*p* < 0.001), C15:0 (*p* < 0.001), C17:0 (*p* < 0.001), C20:0 (*p* = 0.001), C21:0 (*p* < 0.001) and C24:0 (*p* = 0.019). Moreover, TM oil supplementation reduced the level of most MUFAs, such as C14:1 (*p* < 0.001), C16:1 (*p* < 0.001), C18:1 *c*9 (*p* < 0.001), C18:1 *c*11 (*p* < 0.001), C22:1 n-9 (*p* < 0.001) and C24:1 (*p* = 0.003). The PUFA content was also affected by the dietary treatments, and TM oil supplementation showed the highest values for C18:2 c9c12 (*p* < 0.001), C18:3 c9c12c15 (*p* < 0.001) and C20:5 n-3 (*p* < 0.001) and the lowest values for C18:3 n-6 (*p* = 0.002), C20:2 (*p* < 0.001) and C22:2 (*p* < 0.001).

Moreover, the SFA and UFA profiles of the breast tissue were not affected by any of the dietary fat sources (*p* > 0.05). However, TM oil supplementation reduced MUFA content (*p* < 0.001) and increased PUFA content (*p* < 0.001). Moreover, n-3 and n-6 fatty acids were significantly increased with TM oil supplementation (*p* = 0.006; *p* < 0.001, respectively). Additionally, the n6-/n-3 ratio was not affected, but a tendency was observed (*p* = 0.070), and the lowest values were observed with TM oil and poultry fat supplementation. The LNA/LA ratio was affected by dietary fat (*p* < 0.001), and palm oil treatment showed the lowest value. TM oil supplementation decreased Δ9 (14:1/14:0) (*p* = 0.007) and Δ9 (18:1/18:0) (*p* < 0.001). No differences in the thrombogenic and atherogenic indexes were noted (*p* > 0.05)

## 4. Discussion

The fatty acid profile results of the TM oil used in this experiment were dominated by oleic and linoleic acids. These results are consistent with other studies, e.g., Tzompa-Sosa et al. [29], Paul et al. [12], Sosa and Fogliano [13], and Kierończyk et al. [8]. Comparing TM oil with the other fat sources used in this experiment (i.e., poultry fat and palm oil) showed that TM oil had the lowest values of SFAs, whereas palm oil had the highest. UFAs were highest in TM oil and poultry fat and lowest in palm oil. These findings are in agreement with the study conducted by Sosa and Fogliano [13].

In general, the growth performance of broiler chickens did not differ among all the treatments in all periods of the present experiment. According to the obtained results, Kierończyk et al. [8] used *T. molitor* oil and *Z. morio* oil to totally replace soybean oil and did not observe any adverse effects on the growth performance of broiler chickens until they reached 35 days of age. Furthermore, Schiavone et al. [6,7] investigated a total or partial replacement of soybean oil by *H. illucens* oil and did not notice any negative effects on growth performance. All the above studies emphasised that insect oils can be an alternative to soybean oil. Therefore, our study, in which a total replacement of palm oil and poultry fat was performed, corresponded with the findings of Kierończyk et al. [8] and Schiavone et al. [6,7]. Fat sources and their FA profiles are key factors in the growth performance of broiler chickens [30]. For instance, some of the FAs are classified as essential for poultry, because birds are not able to synthesise or convert compounds from other FAs; therefore, they must be supplemented in their diets [1,31]. According to Baião and Lara [32] and Ravindran et al. [1], the essential fatty acids are 18:2 n-6 (linoleic, LA) and 18:3 n-3 (α-linolenic acid, LNA), and their deficiency may retard growth performance. Balnave [33] also stated that a deficiency in linoleic acid may cause decreased growth and low resistance to diseases. In the current study, TM oil had the highest levels of essential FAs, i.e., linoleic acid (C18:2) and linolenic acid (C18:3), followed by poultry fat and palm oil, respectively. Therefore, TM oil can be a new source of fat in broiler chicken diets to replace palm oil and poultry fat. Tancharoenrat et al. [15] reported that the digestibility and absorption of fats depend on their sources. Moreover, it is well reported that the fat of young birds contains high proportions of unsaturated fatty acids that are highly digested, and such fat contains high amounts of saturated fatty acids [30,32]. Regardless of the fat sources, in our study, there were no significant differences among treatments on the apparent ileal digestibility of crude fat, crude protein and AME_N_. These results are in contrast with those of Tancharoenrat et al. [15], who showed that soybean oil and poultry fat were more highly digested then palm oil and tallow, which was explained by the difference in the content of unsaturated fatty acids among fats that are better digested by poultry. Interestingly, in the present study, the fat sources were not comparable in the case of saturated and unsaturated fatty acids; TM oil showed the highest concentration of unsaturated fatty acids. These findings could be explained by the results reported by Sosa and Fogliano [13], who placed insect oils between vegetable oils and animal fats based on the FA profile, with the exception of palm oil, which is comparable to animal fat. According to Józefiak et al. [30,34], the digesta pH values can be affected by dietary sources in different parts of the gastrointestinal tract (crop, jejunum and ceca). However, in our experiment, the pH value was not affected. Furthermore, no effects on lipase and trypsin activity were noted. However, amylase activity was lower in the case of TM oil supplementation. Changes in amylase activity could be explained by a long-term adaptive response of the pancreas to the increased lipid content in the composition of the diet. Experiments performed on rats showed that higher consumption of lipids decreased the intracellular amylase activity and the activity of the amylase secreted from the pancreas [35]. Moreover, some studies indicate that fatty acid composition in the diet can affect α amylase activity. Additionally, experiments performed on diabetic rats have shown that unsaturated fatty acids, such as oleic acid, linoleic acid and palmitoleic acid, and saturated fatty acids, such as stearic acid and palmitic acid, reduce amylase activity [36]. On the other hand, some research did not show any effect of dietary lipid content on amylase activity [37]. Based on this knowledge, we suppose that fat addition and the fat composition of the diet could modulate amylase activity. However, knowledge about this phenomenon is limited and requires further research.

From a physiological point of view, the liver is a vital organ due to its commitment to fat metabolism. Insect oil used in the present study reduced the mass of the liver compared with palm oil and poultry fat. Furthermore, the concentration of triglycerides and total cholesterol in the liver was also lowered by TM oil supplementation in comparison with the other treatments. According to Santoso et al. [38], the reduction in hepatic triglycerides is known to be beneficial for chickens and consumers, as the liver is considered to be an edible chicken organ among many consumers worldwide [39]. It is well known that PUFAs inhibit hepatic lipogenesis, which is the process by which acetyl-CoA is converted to triglycerides [31,40]. In our study, TM oil had the highest amount of PUFAs. Therefore, the low concentration of hepatic triglycerides could be related to the PUFA content in the diet. Fat sources added to broiler chicken diets can change the concentrations of triglycerides in blood [41,42,43]. Serum triglyceride concentrations were reduced more significantly in birds fed TM oil compared with those fed palm oil and poultry fat. A low concentration of serum triglyceride has been observed in chickens after the replacement of dietary SFAs by PUFAs [42,44]. Generally, fats high in SFAs increase the levels of triglyceride in the blood [42]. According to the obtained results, TM oil had the lowest concentration of SFAs and the highest concentration of PUFAs compared with palm oil and poultry fat.

The liver is classified as a by-product in the poultry industry and is consumed on a large scale worldwide [39]. Currently, consumers are more concerned about what they eat, especially regarding nutritional aspects. Some of the most important nutritional aspects are the lipid content and FA profile. It is well documented that the lipid contents and fatty acid profiles of broiler chicken meat and livers can be altered by the feed intake of the birds [45,46,47,48]. Changes in FA profiles, especially decreased SFAs and increased PUFAs, would alleviate criticism by improving nutritional value and would play a key role in improving quality [49]. Our results show that the liver FA profile was affected by the dietary intake of the birds, which is in line with other findings. TM oil supplementation significantly reduced the SFA content, increased the UFA content and, in particular, increased the PUFA content. In addition, TM oil supplementation enhanced the ratio of PUFAs to SFAs, which has a beneficial effect on the nutritional value of the liver. The same findings were reported by Kierończyk et al. [8], who showed that SFAs were decreased and UFAs were increased by use of the oil of both insect species (*T. molitor* and *Z. morio*) in comparison with soybean oil. According to Sim and Qi [50], the synthesis of SFAs can be considerably inhibited in the liver during the digestion of unsaturated fats compared with that of saturated fats. Our results showed that TM oil had the highest amount of PUFAs and the lowest amount of SFAs, which could explain the decreased content of SFAs caused by TM oil supplementation. Furthermore, in our findings, MUFAs were significantly reduced by TM oil supplementation, which could be explained by the increases in PUFA concentrations that suppress the synthesis of MUFAs. According to Pinchasov and Nir [31], PUFAs can inhibit the synthesis of MUFAs through the inhibition of 9-desaturase enzyme complex activity, which is the main enzyme responsible for the conversion of SFAs to MUFAs. Long-chain n-3 fatty acids are well known for their health benefits to humans and animals, such as lowering circulating cholesterol concentrations and reducing the risk of heart disease [47]. Notably, TM oil supplementation significantly increased the n-3 and n-3 PUFA concentrations in the liver in comparison with palm oil and poultry fat supplementation. Furthermore, the n6/n3 ratio was lowest as a result of the TM oil treatment. These findings are in disagreement with the findings of Kierończyk et al. [8], in which decreasing n-3 and n-3 PUFA contents and increasing values of the n-6/n-3 ratio were observed. In a healthy diet, the atherogenic index and the thrombogenic index are recommended to be low [26]. TM oil supplementation reduced both indexes in comparison with palm oil and poultry fat supplementation. The same findings were reported by Kierończyk et al. [8]. Furthermore, the fat content in the liver tissue was affected by dietary fat, and it was significantly reduced by TM oil supplementation. This factor is considered to be a positive change as chicken liver is commonly consumed worldwide. Villaverde et al. [51] reported that increasing the dietary PUFA/SFA ratio can decrease fat deposition in various tissues. Of our dietary fat treatments, TM oil supplementation had the highest level of PUFAs/SFAs, which could explain the low liver fat content in the TM oil-treated group. These findings suggest that the fatty acid composition in the liver tissue was enhanced by the supplementation of TM oil in the broiler chicken diet.

Chicken meat has a high-protein and low-fat content and has been designated as the principal source of PUFAs with a predominant concentration of n-3 PUFAs [52]. Foods with higher PUFA concentrations are considered functional and beneficial for the prevention of coronary heart disease and other chronic diseases [53]. Furthermore, fatty acids play a significant role in the health status of humans, and long-chain fatty acids (LCFAs) are beneficial for the prevention of metabolic disorders [47,48,54]. The present study shows that the saturated and unsaturated fats in the breast muscle tissue were not affected by the dietary treatments used here. However, PUFA concentrations were higher and MUFA concentrations were lower in the case of TM oil supplementation. These results are considered positive as PUFAs are a favourable component of food for human consumption. As reported by Schiavone et al. [6], *H. illucens* oil supplementation in the broiler chicken diet increased SFAs and lowered PUFAs in the breast muscle, and no effects were observed on MUFAs. Furthermore, Kierończyk et al. [8] showed an increase in MUFAs by using supplements of both insect oils (*T. molitor* and *Z. morio*), respectively, but only TM oil supplementation increased the PUFA content in the breast muscle. As mentioned above, the beneficial impact of n-3 and n-3 PUFAs on human health and their added value to food for human consumption were investigated. TM oil supplementation increased the levels of n-3 and n-3 PUFA, as well as n-6 and n-6 PUFA. However, no changes in the n-6/n-3 ratio were observed. These results are not in line with the findings of Kierończyk et al. [8] and Schiavone et al. [6]. The disagreement among the findings of other trials using the same species of insect could be related to the variability of the fatty acid composition of the insect fat due to the rearing conditions at the larval stage [55]. The PUFA/SFA ratio, n-6/n-3 ratio, atherogenic index and thrombogenic index are nutritional indexes that display favourable human health-promoting values in the meat of chickens. However, in our results, the aforementioned indexes were not affected by the dietary treatments.

The two most common PUFAs are linoleic and α-linolenic acids, which act as the primary precursors of n-6 PUFAs [31]. Moreover, LA and LNA are termed essential fatty acids, because they cannot be synthesised by the animal [1,32]. Both fatty acids were significantly enhanced by TM oil supplementation. As mentioned above, TM oil has the highest content of LNA, followed by poultry fat. The LNA content of poultry meat can be improved by supplementing the diet with feed rich in LNA [56,57,58]. Eicosapentaenoic acid (EPA, 20:5n-3) was found to be higher in the breast meat of chickens who ate a diet supplemented with TM oil in the present study. Such fatty acids are important for human health, including brain function and vision. In general, TM oil supplementation in the broiler chicken diet improved the lipid profile of breast meat.

## 5. Conclusions

*Tenebrio molitor* oil as a dietary fat source for broiler chickens was evaluated for its effects on growth performance, nutrient digestibility and liver tissue and breast muscle fatty acid profiles. No adverse impacts on growth performance or nutrient digestibility were observed. Moreover, the TM oil used in this experiment showed promising results by decreasing the fat content in the liver tissue, as well as hepatic triglycerides and total cholesterol. Generally, TM oil supplementation improved the fatty acid profile of the liver tissue and the breast muscle. Therefore, the results of the current study suggest that fat sourced from *T. molitor* larvae may be an alternative to palm oil in broiler chicken nutrition.

## Figures and Tables

**Table 1:** Basal diet composition and nutritional value.

Components (g/kg)	
Corn	546.22
Soybean meal	359.66
Di-calcium phosphate	24.35
Limestone	5.59
TiO_2_	3.0
Premix vit-min ^a^	2.84
DL- Methionine	2.75
Salt NaCl	2.27
L-Lysine	1.80
Sodium Sulphate (Na_2_SO_4_)	1.24
Tryptophan	0.28
**Calculated nutritive value (g/kg)**	
ME (MJ/kg)	11.6
Crude protein	228.0
Crude fat	27.0
Crude fibre	27.5
Na	1.5
Ca	9.8
P total	8.5
P available	4.9
Lysine	13.7
Methionine	5.9
Methionine + Cysteine	9.6
Threonine	8.6

^a^ Premix provided per 1 kg of diet: vitamin A, 11,166 IU; vitamin D_3_, 2500 IU; vitamin E, 80 mg; menadione, 2.50 mg; vitamin B_12_, 0.02 mg; vitamin B_9_, 1.17 mg; choline, 379 mg; D-pantothenicacid, 12.50 mg; vitamin B₂, 7.0 mg; niacin, 41.67 mg; vitamin B_1_, 2.17 mg; vitamin B_7_, 0.18 mg; vitamin B_6_, 4.0 mg; ethoxyquin, 0.09 mg; Mn (MnO_2_), 73 mg; Zn (ZnO), 55 mg; Fe (FeSO_4_), 45 mg; Cu (CuSO_4_), 20 mg; I (CaI_2_O_6_), 0.62 mg; and Se (Na_2_SeO_3_), 0.3 mg.

**Table 2:** Fatty acid profile of palm oil, poultry fat and *Tenebrio molitor* dietary oils (g/100 g fatty acid (FA).

Item	PF ^1^	PO ^2^	TM ^3^
SFA ^5^			
C8:0 Caprylic	0.02	0.02	0.19
C10:0 Capric	0.07	0.02	0.14
C12:0 lauric	1.43	0.22	
C14:0 Myristic	1.22	0.98	2.13
C16:0 Palmitic	20.42	39.57	16.4
C18:0 Stearic	6.11	3.91	2.51
C20:0 Arachidic	0.18	0.00	0.25
MUFA ^7^			
C16:1 Palmitoleic	4.21	0.17	0.76
C18:1 *c*9 Oleic	35.19	41.09	43.8
C18:1 *c*11 Elaidic	3.18	1.24	0.98
C20:1t	0.13	0.35	0.30
PUFA ^8^			
C18:2 *c*9*c*12 Linoleic, LA	23.91	11.19	30.2
C18:3 *c*9*c*12*c*15 α-Linolenic, LNA	1.45	0.29	1.59
**Others ^4^**	0.17	0.18	0.94
SFA ^5^	29.92	44.98	21.6
UFA ^6^	70.07	55.02	78.4
MUFA ^7^	43.51	43.20	46.6
PUFA ^8^	24.79	11.52	31.8
MCFA ^9^	27.59	40.99	19.38
n-6	24.98	11.51	30.2
n-3	1.57	0.48	1.59
n6/n3	15.92	23.99	19.2
PUFA/SFA	0.89	0.26	1.47
Linolenic/Linoleic	0.06	0.03	0.05

^1^ PF—poultry fat; ^2^ PO—palm oil; ^3^ TM—*Tenebrio molitor* oil; ^4^ Othres—C18:1 *c*12, C18:1 *c*15, C20:1 *trans*; ^5^ SFA—saturated fatty acids; ^6^ UFA—unsaturated fatty acids; ^7^ MUFA—monounsaturated fatty acids; ^8^ PUFA—polyunsaturated fatty acids; ^9^ MCFA—medium-chain fatty acids.

**Table 3:** The effect of selected fat sources on the growth performance of broiler chickens.

Treatments	Performance											
	7–14 d			15–21 d			22–30 d			7–30 d		
	BWG, g	FI, g	FCR, g:g	BWG, g	FI, g	FCR, g:g	BWG, g	FI, g	FCR, g:g	BWG, g	FI, g	FCR, g:g
PF ^1^	294	381	1.30	535	772	1.45	813	1309	1.61	1642	2462	1.50
PO ^2^	297	376	1.28	545	763	1.41	833	1310	1.58	1675	2448	1.46
TM ^3^	303	374	1.24	541	760	1.40	798	1293	1.62	1642	2427	1.48
SEM ^4^	22.19	15.66	0.10	34.32	35.65	0.09	40.58	40.71	0.07	63.85	69.27	0.05
*p*-value	0.613	0.474	0.344	0.758	0.694	0.371	0.124	0.553	0.244	0.363	0.474	0.206

^1^ PF—poultry fat; ^2^ PO—palm oil; ^3^ TM—*Tenebrio molitor* oil; ^4^ SEM—pooled standard error of the mean.

**Table 4:** Weights of selected internal organs in relation to the live body weight of broilers.

Treatment	Organ Involvement in Body Weight (% of BW)	
	Liver	Pancreas
PF ^1^	2.42 ^a^	0.22
PO ^2^	2.28 ^ab^	0.24
TM ^3^	2.16 ^b^	0.22
SEM ^4^	0.34	0.04
*p*-value	0.033	0.571

^a–b^ Means within a column with no common superscripts differ significantly (*p* < 0.05);^1^ PF—poultry fat; ^2^ PO—palm oil; ^3^ TM—*Tenebrio molitor* oil; ^4^ SEM—pooled standard error of the mean.

**Table 5:** Changes in amylase, lipase and trypsin activities in the duodenum content after supplementation with different fat sources (differences are expressed as % of control).

Treatments	Enzymes		
	Lipase	Amylase	Trypsin
PF ^1^	100.00	100.00 ^a^	100.00
PO ^2^	119.55	53.23 ^ab^	78.60
TM ^3^	119.12	36.14 ^b^	125.41
SEM ^4^	59.12	55.27	56.34
*p*-value	0.695	0.034	0.197

^a–b^ Means within a column with no common superscripts differ significantly (*p* < 0.05); ^1^ PF—poultry fat; ^2^ PO—palm oil; ^3^ TM—*Tenebrio molitor* oil; ^4^ SEM—pooled standard error of the mean.

**Table 6:** The effect of selected fat sources on the pH value of the gastrointestinal tract content.

Treatments	pH		
	Crop	Jejunum	Caeca
PF ^1^	4.75	5.99	6.60
PO ^2^	5.02	5.98	6.69
TM ^3^	4.83	5.97	6.64
SEM ^4^	0.57	0.26	0.38
*p*-value	0.252	0.983	0.723

^1^ PF—poultry fat; ^2^ PO—palm oil; ^3^ TM—*Tenebrio molitor* oil; ^4^ SEM—pooled standard error of the mean.

**Table 7:** Apparent ileal digestibility of crude protein, crude fat and apparent metabolizable energy corrected to zero nitrogen balance in broiler chickens.

Treatments	Apparent ileal Digestibility		
	CP (%)	CF (%)	AME_N_ (Kcal)
PF ^1^	0.81	0.95	3169.47
PO ^2^	0.78	0.92	3095.66
TM ^3^	0.80	0.92	3116.63
SEM ^4^	0.03	0.04	66.61
*p*-value	0.310	0.360	0.236

^1^ PF—poultry fat; ^2^ PO—palm oil; ^3^ TM—*Tenebrio molitor* oil; ^4^ SEM—pooled standard error of the mean.

**Table 8:** The effect of selected fat sources on the concentrations of selected blood parameters.

Treatments	Blood Parameters						
	NEFA ^4^ (mmol/L)	TP ^5^ (g/dL)	Albumin (g/dL)	Glucose (mg/dL)	Insulin (ng/mL)	TG ^6^ (mg/dL)	TCh ^7^ (mg/dL)
PF ^1^	0.57	4.89	2.91	216.49	0.51	81.11 ^a^	173.04
PO ^2^	0.53	4.67	3.81	215.81	0.65	64.93 ^ab^	169.91
TM ^3^	0.52	4.74	3.10	221.66	0.48	60.52 ^b^	162.98
SEM ^8^	0.08	0.40	1.35	13.67	0.20	19.30	17.93
*p*-value	0.223	0.420	0.248	0.566	0.138	0.040	0.423

^a–b^ Means within a column with no common superscripts differ significantly (*p* < 0.05); ^1^ PF—poultry fat; ^2^ PO—palm oil; ^3^ TM—*Tenebrio molitor* oil; ^4^ non-esterified fatty acid; ^5^ total protein; ^6^ triglycerides; ^7^ total cholesterol; ^8^ SEM—pooled standard error of the mean.

**Table 9:** The concentrations of selected lipid parameters and glycogen in the liver (mg/g of wet tissue).

Treatments	Glycogen	TG ^4^	TCh ^5^
PF ^1^	20.48	9.59 ^a^	3.47 ^ab^
PO ^2^	17.54	9.93 ^a^	3.67 ^a^
TM ^3^	19.21	7.27 ^b^	3.29 ^b^
SEM ^6^	6.26	3.58	0.46
*p*-value	0.337	0.049	0.048

^a–b^ Means within a column with no common superscripts differ significantly (*p* < 0.05); ^1^ PF—poultry fat; ^2^ PO—palm oil; ^3^ TM—*Tenebrio molitor* oil; ^4^ triglycerides; ^5^ total cholesterol; ^6^ SEM—pooled standard error of the mean.

**Table 10:** The effect of selected oils on the summarised fatty acid profile in liver tissue (g/100 g of FA).

Item	PF ^1^	PO ^2^	TM ^3^	SEM ^12^	*p*-Value
SFA ^4^	43.97 ^a^	43.83 ^a^	42.63 ^b^	1.45	0.004
UFA ^5^	56.03 ^b^	56.17 ^b^	57.37 ^a^	1.45	0.004
MUFA ^6^	32.99 ^a^	31.57 ^a^	22.21 ^b^	6.62	<0.001
PUFA ^7^	23.04 ^b^	24.60 ^b^	35.16 ^a^	6.65	<0.001
n-6	20.64 ^b^	21.93 ^b^	31.30 ^a^	5.63	<0.001
n-3	2.14 ^b^	2.17 ^b^	3.58 ^a^	1.05	<0.001
n-6/n-3	10.55 ^ab^	11.22 ^a^	9.27 ^b^	2.22	0.013
n-6 PUFA	20.64 ^b^	21.93 ^b^	31.31 ^a^	5.63	<0.001
n-3 PUFA	2.14 ^b^	2.17 ^b^	3.58 ^a^	1.05	<0.001
PUFA/SFA	0.53 ^b^	0.56 ^b^	0.83 ^a^	0.16	<0.001
LNA/LA	0.02 ^a^	0.01 ^b^	0.01 ^b^	<0.01	<0.001
Total C18:1	29.88 ^a^	28.78 ^a^	19.92 ^b^	6.22	<0.001
MCFA ^8^	26.11 ^a^	24.85 ^a^	19.52 ^b^	3.69	<0.001
LCFA ^9^	73.89 ^b^	75.15 ^b^	80.48 ^a^	3.69	<0.001
Δ9 (14:1/14:0)	0.14 ^a^	0.12 ^a^	0.07 ^b^	0.05	<0.001
Δ9 (16:1/16:0)	0.08 ^a^	0.07 ^a^	0.05 ^b^	0.02	<0.001
Δ9 (18:1/18:0)	0.60 ^a^	0.57 ^a^	0.47 ^b^	0.09	<0.001
TI ^10^	0.73 ^a^	0.70 ^a^	0.50 ^b^	0.13	<0.001
AI ^11^	0.45 ^a^	0.43 ^a^	0.34 ^b^	0.06	<0.001

^a–b^ Means within a column with no common superscripts differ significantly (*p* < 0.05); ^1^ PF—poultry fat; ^2^ PO—palm oil; ^3^ TM—*Tenebrio molitor* oil; ^4^ SFA—saturated fatty acids; ^5^ UFA—unsaturated fatty acids; ^6^ MUFA—monounsaturated fatty acids; ^7^ PUFA—polyunsaturated fatty acids; ^8^ MCFA—medium-chain fatty acids; ^9^ LCFA—long-chain fatty acids; ^10^ TI—Thrombogenic index; ^11^ AI—Atherogenic index; ^12^ SEM—pooled standard error of the mean.

**Table 11:** The effect of selected oils on the fatty acid profile in liver tissue (g/100 g FA).

Item	PF ^1^	PO ^2^	TM ^3^	SEM ^4^	*p*-Value
**Total lipids (mg/g)**	40.13 ^a^	32.64 ^b^	28.57 ^b^	12.31	0.008
SFA ^5^					
C12:0	0.04 ^b^	0.03 ^b^	0.05 ^a^	0.02	0.001
C14:0	0.51 ^a^	0.44 ^ab^	0.42 ^b^	0.11	0.016
C15:0	0.04 ^b^	0.03 ^b^	0.05 ^a^	0.01	<0.001
C16:0	23.15 ^a^	22.24 ^a^	17.81 ^b^	2.84	<0.001
C17:0	0.15 ^b^	0.14 ^b^	0.24 ^a^	0.05	<0.001
C18:0	18.11 ^b^	18.66 ^b^	21.09 ^a^	2.33	<0.001
C20:0	0.11 ^b^	0.13 ^a^	0.14 ^a^	0.03	0.003
C21:0	0.46 ^b^	0.47 ^b^	0.88 ^a^	0.19	<0.001
C22:0	1.23 ^b^	1.56 ^a^	1.78 ^a^	0.43	<0.001
C23:0	0.15	0.16	0.14	0.06	0.582
C24:0	0.03 ^c^	0.04 ^b^	0.05 ^a^	0.02	<0.001
MUFA ^6^					
C14:1	0.08 ^a^	0.07 ^a^	0.03 ^b^	0.04	<0.001
C15:1	0.14 ^b^	0.19 ^ab^	0.22 ^a^	0.09	0.013
C16:1	2.16 ^a^	1.80 ^a^	0.94 ^b^	0.86	<0.001
C17:1	0.25 ^b^	0.27 ^b^	0.40 ^a^	0.13	<0.001
C18:1 *c*9	28.01 ^a^	25.14 ^a^	18.75 ^b^	6.05	<0.001
C18:1 *c*11	1.79	3.56	1.11	0.22	0.105
C18:1 *c*12	0.008 ^b^	0.008 ^b^	0.012 ^a^	*<*0.01	0.009
C20:1 *trans*	0.10 ^b^	0.13 ^a^	0.14 ^a^	0.03	<0.001
C22:1 n-9	0.03	0.04	0.05	0.02	0.719
C24:1	0.34 ^b^	0.28 ^b^	0.52 ^a^	0.18	<0.001
PUFA ^7^					
C18:2 *c*9-*c*12	13.37 ^b^	13.22 ^b^	18.52 ^a^	2.80	<0.001
C18:3 c9-c12- c15	0.27 ^a^	0.18 ^b^	0.28 ^a^	0.08	<0.001
C18:3 n-6	0.39 ^c^	0.53 ^a^	0.46 ^b^	0.11	<0.001
C20:2	0.27 ^b^	0.51 ^a^	0.29 ^b^	0.14	<0.001
C20:3 n-6	6.78 ^b^	8.00 ^b^	12.19 ^a^	3.11	<0.001
C20:4 n-6	0.03 ^b^	0.04 ^ab^	0.04 ^a^	0.01	0.061
C20:5n3	0.78 ^b^	0.86 ^b^	1.53 ^a^	0.48	<0.001
C22:2	0.07 ^b^	0.12 ^a^	0.08 ^b^	0.03	<0.001
C22:5 n-3	1.06 ^b^	1.13 ^b^	1.74 ^a^	0.59	<0.001
C22:6 n-3	0.03	0.02	0.03	0.01	0.233

^a–b^ Means within a column with no common superscripts differ significantly (*p* < 0.05); ^1^ PF—poultry fat; ^2^ PO—palm oil; ^3^ TM—*Tenebrio molitor* oil; ^4^ SEM—pooled standard error of the mean; ^5^ SFA —saturated fatty acids; ^6^ MUFA—monounsaturated fatty acids; ^7^ PUFA—polyunsaturated fatty acids.

**Table 12:** The effect of selected oils on the summarised fatty acid profile in breast muscle tissue (g/100 g of FA).

Item	PF ^1^	PO ^2^	TM ^3^	SEM ^12^	*p*-Value
SFA ^4^	31.38	31.54	31.85	4.51	0.934
UFA ^5^	68.62	68.46	68.15	4.51	0.934
MUFA ^6^	37.65 ^b^	40.77 ^a^	35.29 ^c^	3.16	<0.001
PUFA ^7^	30.97 ^b^	27.68 ^c^	32.86 ^a^	2.61	<0.001
n-6	27.21 ^b^	24.35 ^c^	28.73 ^a^	2.45	<0.001
n-3	3.58 ^ab^	3.01 ^b^	4.10 ^a^	1.16	0.006
n-6/n-3	7.69	8.43	7.65	1.40	0.070
n-6 PUFA	27.21 ^b^	24.35 ^c^	28.73 ^a^	2.45	<0.001
n-3 PUFA	3.58 ^ab^	3.01 ^b^	4.10 ^a^	1.13	0.006
PUFA/SFA	1.03	0.96	1.04	0.36	0.697
LNA/LA	0.045 ^a^	0.036 ^c^	0.041 ^b^	0.01	<0.001
Total C18:1	30.77 ^b^	34.53 ^a^	29.34 ^b^	3.28	<0.001
MCFA ^8^	23.80	25.05	23.54	5.04	0.544
LCFA ^9^	76.20	74.95	75.88	5.09	0.679
Δ9 (14:1/14:0)	0.13 ^a^	0.10 ^ab^	0.08 ^b^	0.05	0.007
Δ9 (16:1/16:0)	0.20	0.21	0.11	0.24	0.291
Δ9 (18:1/18:0)	0.73 ^b^	0.78 ^a^	0.73 ^b^	0.05	<0.001
TI ^10^	0.46	0.52	0.45	0.14	0.208
AI ^11^	0.30	0.32	0.32	0.08	0.504

^a–b^ Means within a column with no common superscripts differ significantly (*p* < 0.05); ^1^ PF—poultry fat; ^2^ PO—palm oil; ^3^ TM—*Tenebrio molitor* oil; ^4^ SFA—saturated fatty acids; ^5^ UFA—unsaturated fatty acids; ^6^ MUFA—monounsaturated fatty acids; ^7^ PUFA—polyunsaturated fatty acids; ^8^ MCFA—medium-chain fatty acids; ^9^ LCFA—long-chain fatty acids; ^10^ TI—thrombogenic index ^11^ AI—atherogenic index; ^12^ SEM—pooled standard error of the mean.

**Table 13:** The effect of selected oils on fatty acid profile in breast muscle tissue (g/100 g FA).

Item	PF ^1^	PO ^2^	TM ^3^	SEM ^4^	*p*-Value
**Total lipids (mg/g)**	10.71	11.13	11.70	4.340	0.7284
SFA ^5^					
C12:0	0.19 ^a^	0.09 ^b^	0.10 ^b^	0.03	<0.001
C14:0	0.55 ^b^	0.52 ^b^	0.84 ^a^	0.09	<0.001
C15:0	0.08 ^b^	0.06 ^c^	0.11 ^a^	0.02	<0.001
C16:0	19.53 ^b^	22.41 ^a^	18.85 ^c^	0.81	<0.001
C17:0	0.17 ^b^	0.13 ^c^	0.21 ^a^	0.02	<0.001
C18:0	10.33 ^a^	8,77 ^b^	9.60 ^b^	1,08	<0.001
C20:0	0.19 ^ab^	0.18 ^b^	0.21 ^a^	0.03	0.001
C21:0	0.53 ^b^	0.44 ^c^	0.67 ^a^	0.10	<0.001
C22:0	0.88	0,93	0.85	0.25	0.614
C23:0	0.22 ^a^	0.17 ^b^	0.16 ^b^	0.05	0.002
C24:0	0.05 ^b^	0.05 ^b^	0.07 ^a^	0.03	0.014
MUFA ^6^					
C14:1	0.08 ^a^	0.06 ^b^	0.06 ^b^	0.02	<0.001
C15:1	2.00	2.05	2.01	0.55	0.947
C16:1	2.85 ^a^	2.39 ^b^	1.82 ^c^	0.45	<0.001
C17:1	0.86	0.74	0.79	0.17	0.082
C18:1 *c*9	27.53 ^b^	31.43 ^a^	28.08 ^b^	2,61	<0.001
C18:1 *c*11	2.62 ^a^	2.14 ^b^	1.70 ^c^	0,22	<0.001
C18:1 *c*12	0.01	0.01	0.01	0.01	0.943
C20:1 *trans*	0.09	0.09	0.09	0.01	0.500
C22:1 n-9	0.04 ^a^	0.02 ^b^	0.02 ^b^	0.01	<0.001
C24:1	0.80 ^a^	0.58 ^b^	0.69 ^b^	0.20	0.003
PUFA ^7^					
C18:2 *c*9-*c*12	19.73 ^b^	17.60 ^c^	22.26 ^a^	1.18	<0.001
C18:3 c9-c12- c15	0.90 ^a^	0.64 ^b^	0.90 ^a^	0.14	<0.001
C18:3 n-6	0.34 ^b^	0.38 ^a^	0.33 ^b^	0.04	0.002
C20:2	0.18 ^b^	0.30 ^a^	0.14 ^b^	0.11	<0.001
C20:3 n-6	6.42 ^a^	5.30 ^b^	6.42 ^a^	1.43	0.016
C20:4 n-6	0.04	0.05	0.04	0.03	0.199
C20:5n3	1.78 ^b^	1.54 ^b^	2.16 ^a^	0.46	<0.001
C22:2	0.11 ^b^	0.17 ^a^	0.09 ^b^	0.06	<0.001
C22:5 n-3	0.80	0.68	0.64	0.30	0.193
C22:6 n-3	0.03	0.04	0.03	0.03	0.264

^a–b^ Means within a column with no common superscripts differ significantly (*p* < 0.05); ^1^ PF—poultry fat; ^2^ PO—palm oil; ^3^ TM—*Tenebrio molitor* oil; ^4^ SEM—pooled standard error of the mean; ^5^ SFA —saturated fatty acids; ^6^ MUFA—monounsaturated fatty acids; ^7^ PUFA—polyunsaturated fatty acids.
